# The impact of face masks on interpersonal trust in times of COVID-19

**DOI:** 10.1038/s41598-021-96500-7

**Published:** 2021-08-30

**Authors:** Samreen Malik, Benedikt Mihm, Malte Reichelt

**Affiliations:** 1 Division of Social Science, New York University Abu Dhabi, Abu Dhabi, UAE; 2 Otto-von-Guericke University Magdeburg, Magdeburg, Germany

**Keywords:** Human behaviour, Statistics

## Abstract

Despite the widespread use of face masks to combat COVID-19, little is known about their social and behavioral consequences. To understand the impact of face masks on interpersonal trust, we designed a novel experiment to assess the causal impact of face mask use on whether individuals follow economically relevant advice from a stranger. From a survey of more than 2000 US citizens, conducted during July and August 2020, we find that almost 5% fewer individuals trust advice when it is given by someone wearing a mask than when it is given by someone not wearing a mask. While, surprisingly, health-related risks do not seem to alter the way masks affect trust, the effects of masks are particularly large among individuals whose households face economic risks due to COVID-19 and those with below-average normative beliefs about mask wearing. Our results highlight the non-health-related meaning that face masks have developed during COVID-19 and suggest that mask use undermines trust in others among a substantial share of the US population.

## Introduction

In July 2020, the World Health Organization (WHO) Director General stated that the COVID-19 pandemic constitutes the “most severe global health emergency ever declared by the WHO”^1^. As part of the preventive measures to curb the spread of the virus, many governments and health officials have advised or mandated the use of face masks in public spaces^2^. Numerous studies have analyzed and highlighted the health-related consequences of mask use^3–6^. However, although face masks have been part of nearly every interaction in public spaces, little is known about the social and behavioral consequences of their use. Because of the non-health-related meaning that face masks have developed^7^ and diverging attitudes around preventive measures^8^, we argue that their widespread use affects and redefines who trusts whom in today’s society. Such interpersonal trust is a central pillar of cohesion in societies^9^ because it influences cooperative behavior, reduces conflict, and increases group and economic performance^10,11^.

Whether mask use increases or decreases interpersonal trust on aggregate is unclear. On the one hand, wearing masks can signal prosocial behavior^12^, which should increase perceived trustworthiness^13^. For the majority of people, a SARS-CoV-2 infection is uncritical^14^, but many who are infected with SARS-CoV-2 are asymptomatic or presymptomatic and thus not visibly sick^15^. Most of the time, masks thus serve as a means to protect others in the case of an infection^16^. On the other hand, face masks cover large parts of the face, making face recognition difficult^17–19^ and hiding expressions that are important to evaluate trustworthiness and establish trust^20,21^. Moreover, masks have become a controversial and politically charged topic^22,23^. Attitudes around preventive measures differ strongly even within societies^8^, and a mask may signal membership to an opposing ideological group^24^, which decreases perceived trustworthiness, particularly when individuals are not easily identifiable^25^. The decision to wear a mask should thus evoke fundamentally different reactions among members of society and redefine who trusts whom. As the mask’s connotation as prosocial or as an ideological symbol should be attached to potential personal consequences of COVID-19 and to one’s beliefs and attitudes, we explicitly analyze their effects conditional on belonging to a risk group and on personal normative beliefs about mask wearing.

*COVID-related risks:* Risks associated with COVID-19 are unequally distributed. In particular, older individuals and those with underlying health conditions face a greater mortality risk^14,26^. In terms of trust, we thus predict that wearing a mask evokes either a more positive or a less negative response among older and unhealthier individuals. The pandemic, however, also introduced the risk of experiencing severe economic consequences. This risk is particularly high for individuals who themselves or whose partner lost their job or had to reduce their working hours^27–29^. As the likelihood of facing negative economic consequences is also unequally distributed, wearing masks (and thus potentially contributing to a quicker economic recovery) may again signal prosocial behavior to those at an increased risk of economic consequences. However, those with COVID-19-related economic worries have also been shown to hold more negative attitudes towards preventive measures^30^. Wearing face masks might then be less associated with prosocial behavior and more associated with political decisions that caused their personal economic hardship. In terms of trust, wearing masks might thus evoke both more positive or negative reactions among those facing high economic risks.

*Personal normative beliefs:* While assumed personal risks should affect the perception of masks, beliefs about masks’ effectiveness, an aversion to being forced to wear masks, or political ideologies also shape individual attitudes about face masks^22,23^ and may thus further define the effect of mask use on trust. Although wearing a face mask in public spaces or when meeting others is an emerging norm^31^, personal normative beliefs about when and where one should wear a face mask should thus differ quite substantially. The decision to wear a mask (at least when not externally enforced) makes one’s beliefs about what should be done visible to others. As individuals tend to trust others less when they hold diverging normative beliefs and values^32–34^, we thus predict that in terms of trust, wearing a mask evokes a more negative (or less positive) response from individuals with weaker or negative personal normative beliefs toward mask wearing.

Aiming to causally test how face mask use affects interpersonal trust for various groups in society imposes multiple challenges. We first need to provide a large and representative sample of participants with a controlled environment in which they are exposed to an unknown person they may trust or distrust. This person randomly needs to wear or not wear a face mask. Wearing a mask should appear reasonable, but not mandatory, so that the mask is seen as a choice and can thus be interpreted as a prosocial act or an ideological statement. This treatment should be subtle to avoid potential experimental demand effects that may confound the results.

**Figure 1:**
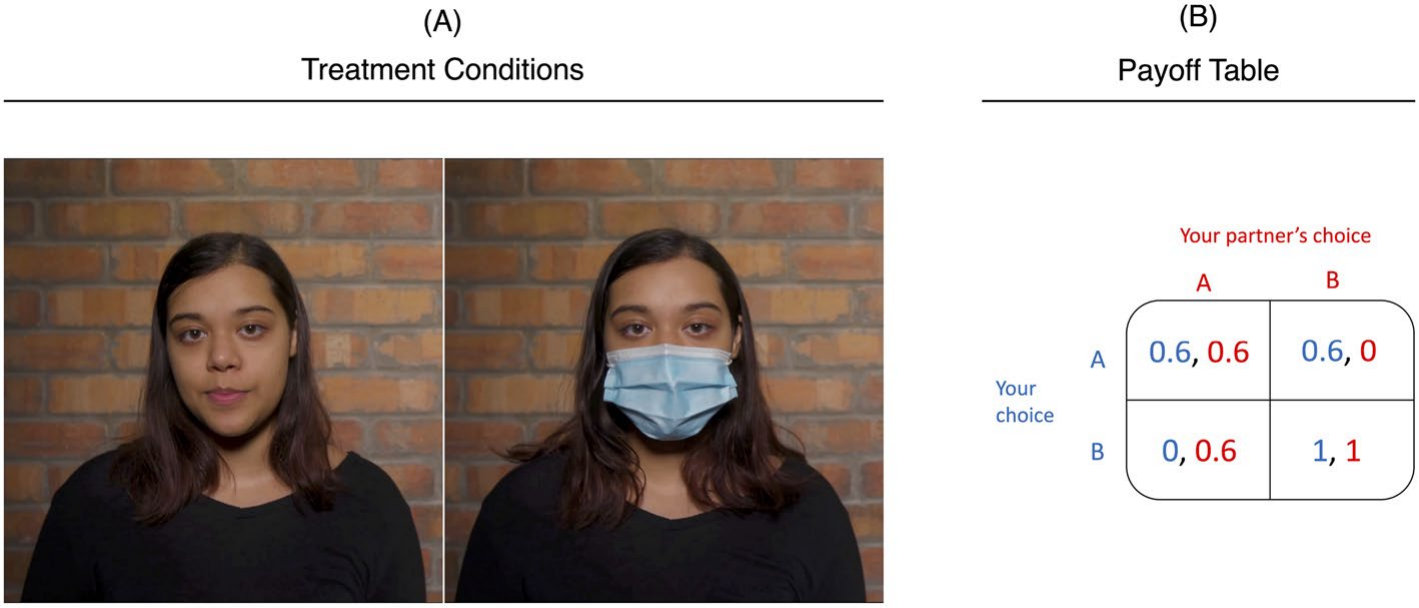
Experimental setup. *Note*: (**A**) shows a video snapshot of the two randomized treatment conditions; (**B**) shows the payoff matrix for the game where the first value in each cell represents the payoff in US dollars the participant receives, conditional on the opponent’s choice.

We address these challenges by designing a novel online survey experiment in which we present a representative sample of 2300 US citizens with a simple decision problem that offers them the opportunity to substantially increase their survey participation fee, providing advice that they can decide to follow or not, which allows us to measure trust. Playing a one-shot economic game (stag hunt), participants are given two possible choices. The outcome of the game, and thus the potential payoff, depends on their own and their opponent’s choice, leaving no clear best strategy. We make use of this ambiguity and allow a masked or unmasked actor to first explain the rules of the game and then provide the participants with advice on how best to solve the decision problem (see Fig. 1, Panel A). Advice and whether the actor wears a mask are randomly assigned across the participant pool. To create a setting in which it seems natural to wear a face mask, we provide the explanation and advice in video format. The backdrop of the video is designed to mimic a public space with noise of lively chatter, making wearing a face mask reasonable but not mandatory. While interpersonal trust has many facets, in the most general sense, it can be defined as a belief about the trustworthiness of another person and thus as a belief about their capability and motivation to fulfill one’s own interest^35,36^. As such, it is the willingness to be vulnerable to another party^32^. In our setup, we measure such trust with the decision to follow or not follow a stranger’s suggestion in a personally relevant economic decision. Measuring trust in response to wearing a mask allows us to assess potential social and behavioral consequences of the increased use of face masks.

While the outcome of the economic game is irrelevant for our study, we choose a stag hunt game because it does not provide a unique optimal strategy (see Fig. 1, Panel B). It is therefore well suited to including reasonable suggestions of how best to play the game (Pareto dominance vs. risk dominance). We randomly select a small number of the participants to whom we do not show the video and do not provide advice. We thus neither assign them to the treatment nor the control group, but select these participants as real opponents in the game, which creates a realistic setting for the remaining participants, who are assigned to our experimental conditions. We reassure the participants in the control and treatment group that their opponents will not receive advice to ensure that the participants do not change their assumptions about the most likely choice of their opponent (see “Methods” section for more details on the experimental design and the Online Supplement for the transcript of the video).

## Results

### General effect of masks on trust

**Figure 2:**
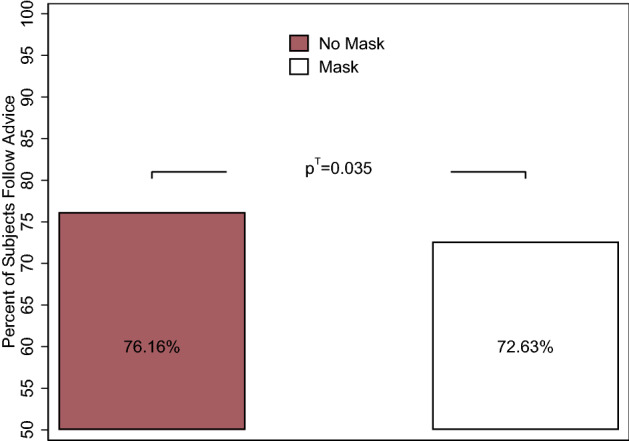
Following advice. *Notes*: The bar in red depicts the unconditional mean in the control (no mask) condition ($N=906$), and the bar in white depicts the mean in the treatment (mask) condition ($N=961$). The $p^T$-value represents the *p*-value for the treatment effect. Standard errors are clustered at the state level. Conditional estimates controlling for state fixed effects, week fixed effects, design controls, and individual groups are presented in Table S4.

Are individuals overall less trusting of others who wear face masks? In Fig. 2, we display our main treatment effect, showing how likely individuals in our sample are to follow the advice given in the preceding video. These—and all the following—results are unconditional, meaning they provide the raw averages across our sample, without controlling for additional variables. We re-estimate all effects controlling for design effects, state and week fixed effects, and individual controls and present the main results in Table 1. Full results, which mirror the findings presented here, and additional robustness analyses using different model specifications, weighting, and alternative measures for our variables of interest can be found in the Online Supplement (Tables S3 and S4). A description of the empirical models can be found in the “Methods” section.

If participants completely ignored the advice, our randomization implies that on average, 50% should follow the actor’s suggestion. Across all participants, however, approximately 74% follow the advice. This high percentage mirrors findings that advice in decision-making generally tends to be followed when the adviser is considered an expert^37^, which in our case is the actor explaining the rules of the game. Relative to individuals who receive advice from a nonmasked actor (control group), individuals who receive advice from a masked actor (treatment group) are 4.63% (approx. 3.5 percentage points) less likely to follow the advice. The effect size is statistically significant ($p^T$-value $=$ 0.035). We thus find that the negative effect of mask use on trust—either through hidden facial expressions or through negative connotations—is on average not outweighed by the positive connotation of masks among some members of society.

As we show in the Online Supplement Fig. S1, the differences in interpersonal trust towards masked or unmasked strangers are not mirrored by differences in generalized trust levels, which confirms that the results are not due to treated individuals being less trusting by chance. We also show that weighting our sample to exactly achieve the targeted representative sample (see Online Supplement Table S1) is not of concern (see Online Supplement Table S2 for DuMouchel and Duncan test) and does not bias our results (see Online Supplement Table S4, column (5)).

**Table 1:** Summary of estimates.

**Treatment effect: mask versus no mask**				
Coefficient (*beta*) (Standard error (*se*))	**Aggregate**	**Health risk**	**Economic risk**	**Normative beliefs**
		***Low***	***Low***	***Below average***
	$${-0.043^{**}}$$ (0.018)	$$-0.070^{**}$$ (0.032)	−0.028 (0.019)	$$-0.087^{***}$$ (0.031)
		***High***	***High***	***Above average***
		−0.020 (0.021)	$$-0.12^{***}$$ (0.042)	0.00048 (0.027)
***Difference between treatment effects:***	-	***Low vs. High***	***Low vs. High***	***Below average vs. Above average***
Coefficient ($beta^D$) (Standard error ($se^D$)) [*p*-value ($p^D$)]	-	0.050 (0.039) [0.20]	-0.091 (0.043) [0.040]	$0.087^{*}$ (0.046) [0.066]

*Notes:* To empirically estimate the treatment effect *T*, we use a linear probability specification: $Y_{is} = \alpha _{s} + \beta T_{is} + \gamma X_{is} + \epsilon _{s}$, where $Y_{is}$ is the dummy for following advice for individual *i* in state *s*. To estimates the difference in treatment effects by group, we interact the group variable of interest with the treatment dummy. Standard errors (in brackets) are clustered at the state level. The stars indicate significance: *${p}<0.100$ **${p}<0.050$, and ***${p}<0.010$. For the main treatment effect, $X_{is}$ includes *design controls*, such as the suggested option during the experiment, and the order of the experiment and the survey questions about normative beliefs; *demographic controls*, such as gender, race, and education; *normative beliefs* and *COVID-related risk covariates*, including the composite index for health risk measured as 1 for older respondents (age greater than 50) or respondents who have reported fair or poor health conditions. Both of these factors mean that this group has a higher health risk and composite index for economic risk, measured by the respondent reporting a transition to unemployment or reduced work hours between January and July/August for the household. For the differences in treatment effects by group, we include all the controls except for the group variable of interest. We also provide the coefficient, standard errors (in brackets), and the p-value ($p^D$) for the difference in treatment effects by group mentioned in the header [in square brackets]. For all the columns, the *sample size = 1867* (with $N=906$ in the control condition and *N* = 961 in the treatment condition). See Appendix Table S4 and Appendix Table S5 for the full tables.

### Treatment effect by COVID-related risk

**Figure 3:**
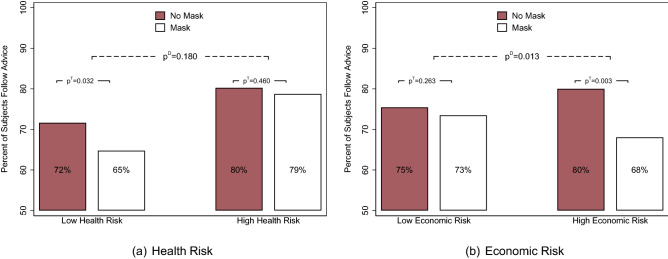
Following advice by risk. *Notes*: The bars in red depict the unconditional mean in the control (no mask) condition ($N=906$), and the bar in white depicts the mean in the treatment (mask) condition ($N=961$). The $p^T$-value represents the *p*-value for the treatment effect, and the $p^D$-value is the *p*-value for the difference across the treatment effects for the mentioned groups on the x-axis. Standard errors are clustered at the state level. Conditional estimates controlling for state fixed effects, week fixed effects, design controls, and individual groups are presented in Table S5.

We next evaluate whether the effect of face mask use on interpersonal trust is less negative or even positive among those at a higher health risk, as we assume that wearing masks might be interpreted as a prosocial act. We therefore divide our sample into participants who are either above the *age* of 50 or who declare their *health* status as fair or poor and those below 50 or with good, very good, or excellent health. We choose age 50 as a threshold, as the COVID-related health risk for individuals below 50 is close to zero^38^. Figure 3a shows that on average, mask use lowers trust among those with a lower health risk: 9.7% (approx. 7 percentage points) fewer participants follow the advice given by the stranger if the stranger is wearing a mask ($p^T$-value = 0.032). Among those with a high health risk, wearing a mask does not substantially alter the probability of following the actor’s advice. A comparison of the treatment effect across the two groups, however, shows that the difference is insignificant ($p^D$-value = 0.180), indicating that mask use does not evoke a substantially more positive reaction among those in the group with a higher health risk. The insignificant difference in treatment effects is robust to analyzing health and age separately as well as analyzing stricter risk groups with older and unhealthier individuals (see Online Supplement Fig. S3(a)–S3(b) and Fig. S4 for more details).

While characteristics that should increase the perceived risk of experiencing health-related consequences of COVID-19 seem to play only a marginal role in whether face masks affect interpersonal trust, economic consequences clearly seem to matter. We again split our sample into two groups. The first group includes individuals who themselves or whose partners became *unemployed* or had to *reduce their work hours* by more than 8 h (one full work day) per week between January and July/August 2020. For this group, we assume a higher risk of experiencing economic hardship due to COVID-19. As Fig. 3b shows, we find that among those at risk of negative economic consequences due to COVID-19, approximately 15% (approx. 12 percentage points) fewer individuals follow the advice given by the stranger if the stranger is wearing a mask ($p^T$-value = 0.003). There is no significant treatment effect among those who did not experience negative economic consequences, and by comparing the effects across the two groups, we find that the difference is significant ($p^D$-value = 0.013). All our results hold in the full empirical model while controlling for other group characteristics (see Table 1). Analyzing unemployment and reductions in hours separately shows that the latter group is mostly responsible for the negative effect of mask use on trust (see Online Supplement Fig. S3(c)–S3(d)). This may be the case because only a few people in our sample have become unemployed but many have become furloughed.

### Treatment effect by personal normative beliefs

**Figure 4:**
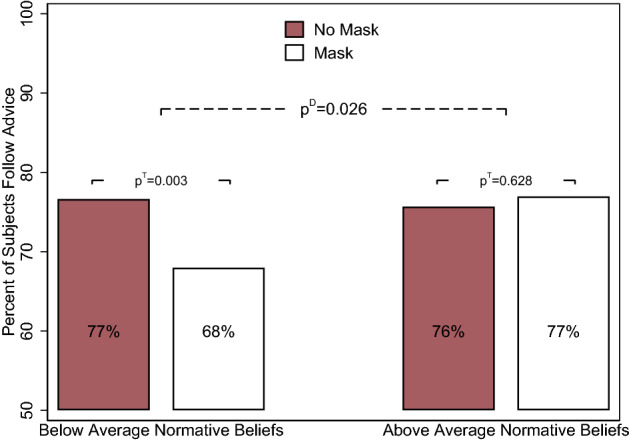
Personal normative beliefs. *Notes*: The bar in red depicts the unconditional mean in the control (no mask) condition ($N=906$), and the bar in white depicts the mean in the treatment (mask) condition ($N=961$). The $p^T$-value represents the *p*-value for the treatment effect, and the $p^D$-value is the *p*-value for the difference across the treatment effects for the groups mentioned on the x-axis. Standard errors are clustered at the state level. Conditional estimates controlling for state fixed effects, week fixed effects, design controls, and individual groups are presented in Table S5.

We now focus on *personal normative beliefs about mask wearing* and characterize individuals by their beliefs about whether one should be wearing a mask in different situations (in enclosed public spaces such as grocery stores, outside, using public transportation, and when meeting people in public). We divide our sample into those with above-average and below-average (median) normative beliefs. We present the results in Fig. 4. For the group with below-average normative beliefs, we observe that 13.23% (approx. 9 percentage points) fewer participants follow the advice given by the stranger wearing a mask ($p^T$-value = 0.003). The effect is insignificant among those with above-average normative beliefs about mask wearing, and the differences in the treatment effects are significant (the $p^D$-value is = 0.026). Participants’ personal normative beliefs about mask wearing thus play a crucial role in the impact of masks on interpersonal trust. The effect of mask use on following advice among those with below-average normative beliefs is approximately three times larger than that among the general population. Interestingly, those with above-average normative beliefs and those with below-average beliefs follow the advice of the unmasked person with approximately the same likelihood.

The results regarding individuals’ personal normative beliefs hold when including all other group indicators (COVID-related risk factors) as control variables (see Table 1), highlighting that own beliefs about when to wear a mask are not simply a function of perceived health-related or economic risks (we re-estimate the effects using all disaggregated and more detailed variables used to construct the risk groups in the Online Supplement; see Figs. S5 and S6). We also show that despite the politicized nature of preventive measures, political ideology is not an important factor driving the treatment effect (see Fig. S7 in Online Supplement). Different normative beliefs about mask wearing thus seem to have more complex origins, cross-cutting political views and factors that should influence the perceived health and economic risks of COVID-19.

## Discussion

Preventive measures to curb the spread of SARS-CoV-2 have extensively changed the way people interact with one another, evoking questions about their social and behavioral consequences. Using a novel experimental design, our study is the first to show that wearing face masks during the pandemic negatively affects interpersonal trust. Approximately 5 percent fewer individuals follow economically relevant advice when it is given by someone wearing a face mask than when it is given by someone not wearing a mask. The impact is non-negligible, as—using 2019 census data—3.5-percentage-points fewer individuals in our sample following advice in the treatment condition translates into roughly 8.8 million more people in the US showing lower trust towards others if they wear a mask. Many situations in which trust is of essential importance resemble our experimental setting, e.g., when advice by politicians or other public figures is being broadcast and face masks are being used (or not being used) for their symbolic value and to establish credibility among the audience.

Surprisingly, those who should perceive their COVID-related health risk as higher—older or unhealthier individuals—do not substantially trust others wearing masks more than others not wearing masks. However, when households face an increased economic risk, 15% fewer individuals trust others wearing masks than others not wearing masks. Moreover, among those with below-average normative beliefs about mask wearing, others seem substantially less trustworthy when they wear masks. More than 13% fewer individuals in this group follow the advice given by the masked stranger than that given by the unmasked stranger. The finding that one’s health risk does not significantly alter the effects of masks on trust but that one’s economic risk and personal normative beliefs matter highlights the strong non-health-related meaning of face masks during the pandemic, in which masks may be interpreted as support for governmental policies or as an ideological statement. The results also emphasize the risk that originates from preventive measures’ unequal economic impact on people’s lives. Curbing the spread of the virus requires a collective effort, but if the costs are shouldered unequally, trust in preventive measures and those supporting these measures may suffer. However, our results show that consequences are not attached only to objective risks and highlight the strong role that heterogeneous normative beliefs around face masks play in the social and behavioral consequences of the pandemic.

In contrast to previous studies, which found slightly positive or null results based on survey-based measures of perceived trustworthiness^19,39,40^, we find robust negative effects on trust. The differences may arise because facial dynamics have been shown to be important for interpreting facial expressions^41,42^, which affect trust^20^. Video (or live) exposure to faces with and without masks might thus evoke different reactions compared to exposure to still images. Moreover, our design introduces a subtle measure of interpersonal trust that does not invite participants to actively think about trust. As previous studies have shown, survey measures on trust and actual behavior are not necessarily related^43^ (see Online Supplement Fig. S2 for nonsignificant findings on additional survey-based measures on trust). Furthermore, our study is the first to test the effects of masks in the US, which represents a country in which attitudes around governmental responses to COVID-19 are particularly heterogeneous^8^.

Our experiment yields further insights. Previous research has shown that trust in others’ behavior depends on beliefs about their adherence to social norms. While the norms previously studied were directly related to behavior (e.g., transaction norms or exchange practices^44^), the COVID-19 pandemic allows us to experimentally evaluate whether normative beliefs about an unrelated issue likewise affect trusting behavior. In our case, a visual cue of a personal normative belief (wearing a face mask when not mandatory) affects individuals’ beliefs about their trustworthiness if they have weaker or negative normative beliefs about mask wearing.

One limitation of our research lies in the experimental setting: we cannot assess whether the effects of masks are different in truly interactional settings, in which the negative effect of a mask could be offset by trust established through mutual communication, for example when interacting with a non-stranger. Nonetheless, the fact that the effects are driven primarily by individuals with below-average normative beliefs about mask wearing and individuals at a higher economic risk suggests that the symbolic meaning of masks plays a strong role in this relationship. In a polarized environment, the interpretation of face masks may thus be particularly difficult to change.

While we are measuring the effects of face masks on trust in economic advice, it is an open question whether the findings extend to other types of advice. For example, in the medical sector, face masks may have a different meaning^45^, signaling professionalism and care for patients. While we would expect trust not to be negatively affected by wearing a mask in these contexts, other studies have shown that interactions between doctors and patients are affected by wearing face masks^46^. It is an open question whether the meanings of masks in the medical context have also changed during the pandemic. Moreover, while we are measuring the effects of medical face masks, which are perhaps one of the most common types of masks used due to recommendations by the CDC, other types of masks (e.g., see-through masks or masks with printed elements on them) could potentially reduce the negative impact of hidden facial expressions. However, we would expect the symbolic meaning of masks to persist and thus to affect interpersonal trust depending on mask-wearing norms, health and economic risks.

The increased usage of face masks and their negative effect on interpersonal trust suggests that during the pandemic, overall trust has likely declined, an assumption that is mirrored in recent empirical findings^47^. While our results highlight the potential unforeseen consequences of the widespread use of face masks, we would like to emphasize that the results do not suggest abandoning them but rather re-establishing and increasing trust, for example, through messaging regarding social norms or the introduction of solidarity mechanisms to cushion the economic consequences of the pandemic^48^. Such efforts will be particularly important because it remains unclear how long preventive measures will be required and masks may continue to be used beyond the pandemic, as has been observed in other contexts^49^.

## Methods

### Experimental design

**Game:** In the main part of the survey, each respondent plays an incentivized stag and hare game^50^ with another randomly selected anonymous respondent. In the game, a respondent chooses between two options, A and B. The payoff from their decision depends on both their own decision and that made by their opponent. The decision of the opponent is not known to the respondent when choosing between the options, and vice versa.

**Figure 5:**
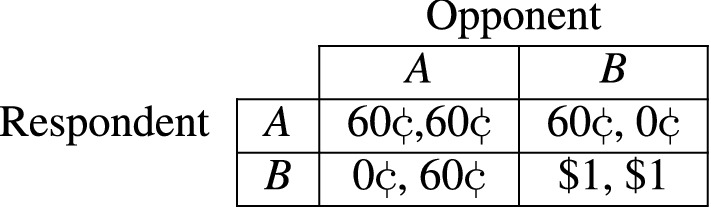
Stag and hare game.

Figure 5 shows the payoff matrix for the game in which the first value in each cell represents the payoff the respondent receives, conditional on the opponent’s choice.

The game has no clear best option for the respondents. In particular, if a respondent thinks the opponent will choose *A*, then they should also choose *A*, and if a respondent thinks the opponent will choose *B*, then they should also choose *B*. Moreover, if both choose *B*, the respondent receives a higher payoff than they would have if both had chosen *A*. However, if the respondent chooses *B* and the opponent chooses *A*, then the respondent receives nothing, whereas if the respondent chooses *A*, they receive 60 ¢ regardless of what the opponent does. Choosing *A* is therefore less risky than choosing *B*, but choosing *B* provides a higher payoff as long as the opponent also plays *B*. Formally, the game has two Nash equilibria (*A*, *A*) and (*B*, *B*), where the equilibrium (*B*, *B*) payoff dominates the equilibrium (*A*, *A*), and the equilibrium (*A*, *A*) risk dominates the equilibrium (*B*, *B*).

A subsample (10%) of respondents was randomly selected and assigned the role of the opponent. The opponent always received the instructions for the game in written form. All other respondents participated in our two conditions in which they received the instructions for the game from an actor in a video.

**Video:** In the video, the actor explains the game in Fig. 5 to the respondents. After explaining the game, the actor provides a suggestion regarding which of the two options (*A* or *B*) the actor would suggest the respondent choose. In particular, the actor either suggests choosing *A* because it is the safer option or *B* because it is the option that provides the higher payoff. Which advice a given respondent receives is random. As the game does not provide a clear superior strategy, the purpose of the advice is to elicit whether the respondent trusts the actor in the video in an incentivized way: if a respondent trusts the actor, they should be more likely to follow the advice.

**Conditions:** There are two conditions that differ only in terms of whether the actor in the video is wearing a mask (treatment) or not (control). Everything else in the videos is identical: the actor, the text, the advice, and the setting. The conditions therefore allow us to test whether trust in the actor depends on whether the actor is wearing a mask by testing whether respondents are more likely to follow the advice in either of the two conditions.

**Ethical considerations:** The study was approved by the institutional review board (IRB) at NYU Abu Dhabi (HRPP-2020-77). All experiments were performed in accordance with relevant guidelines and regulations and after obtaining online informed consent from participants. All participants were at least 18 years of age. Additionally, informed consent was acquired from the actor to include her photograph in the paper and online publication.

### Empirical model

Given the randomized design of our experiment, we estimate linear probability models with a number of covariates to identify the causal impact of interest. To identify the treatment effect of the treatment condition *T* on outcome *Y*, our specification is

1 $$\begin{aligned} Y_{is} = \alpha _{s} + \beta T_{is} + \gamma X_{is} + \epsilon _{s} \end{aligned}$$

where *i* denotes the individual and *s* denotes the US state of residence. Our primary outcome of interest is a binary variable $follow \;advice$, which takes a value of 1 if the individual followed the suggested option in the video and 0 if they deviated from the suggestion provided by the actor, thus measuring trust in a stranger in an economically relevant situation. While our randomization allows for an unbiased estimation of the treatment effect on *Y*, we further improve its precision by including covariates *X* (described below). We also include state fixed effects to ensure that the differences in the rules for combating COVID-19 across states do not bias our results. Since the surveys were conducted over a period of five weeks, we also include week fixed effects to control for any changes in the news cycle from one week to another. Finally, we cluster the standard errors at the state level. As data across states were collected iteratively and not all states were targeted at the same time and state policies or other unobserved factors within states may have changed during the data collection period, the data we collect represent a sample of all theoretically possible relevant state-time combinations over this period of time, which requires accounting for potential serial correlation and heteroskedasticity using clustered standard errors^51,52^.

The covariates *X* are of three types and include (i) design-specific control variables for whether the provided suggestion was option A or B and whether the norm-elicitation questions were collected before or after the experiment, (b) a battery of additional covariates, which include race, gender, and education, and (c) our main independent variables of interest, which include the respondents’ personal normative beliefs regarding mask wearing and their COVID-related risks (health risk and economic risk).

For personal normative beliefs, the respondents are asked to provide their beliefs about wearing masks in certain situations. In particular, the respondents are asked to rate on a scale of $-5$ (strongly disagree) to 5 (strongly agree) whether they believe people should wear a mask in (i) enclosed public spaces such as shops, (ii) when outside, (iii) when using public transport, and (iv) when meeting people in public. The questions on personal normative beliefs are randomly asked either before or after the videos to control for unwanted priming effects. We build an index across all four contexts and create a binary variable taking a value of 1 if the respondent has strong normative beliefs (above median values on the personal normative belief index). The variable takes a value of 0 if an individual holds weak or negative normative beliefs (below median values on the personal normative belief index).

To measure one’s health risk of COVID-19, we construct a composite index that is based on two variables. The first variable measures whether the respondent is above 50 years of *age*. The second variable measures whether the respondent perceives their *own health* to be fair to poor. The composite index takes a value of 1 if either of the risk factors is present; otherwise, it takes a value of 0.

To measure one’s economic risk, we construct a composite index, which is based on four variables. These variables measure whether the respondent or their partner became unemployed or whether they experienced a reduction of working hours of at least one full work day per week ($>8$ h) between the months of January (before the spread of COVID-19 in the U.S.) and July/August (after the introduction of protective measures against COVID-19). If the respondent or their partner experienced such negative labor market-related consequences, we assume an increased economic risk, and the factor takes a value of 1.

To study the difference in our treatment effects by group, we interact the treatment *T* with our main independent variables of interest, which include personal normative beliefs and COVID-related risks (health and economic risk). In the unconditional specification, no controls are included. In the full specification, we control for state fixed effects, week fixed effects, design controls, individual controls, and all main independent variables.

For additional analyses, we also use political ideology as a group characteristic and thus as an interaction term (see Fig. S7) and as a control (see Fig. S6a). We elicit the political ideology of the respondents by asking them whether they classify themselves as liberal or conservative on a scale ranging from extremely liberal to extremely conservative. We subsume the categories to take a value of 1 for liberal, 2 for moderate and 3 for conservative political ideologies.

## Supplementary Information

Supplementary Information 1.
