# Supplementary material for: The impact of face masks on interpersonal trust in times of COVID-19
Source: Sci Rep. 2021 Aug 30;11:17369. doi: 10.1038/s41598-021-96500-7 (PMC8405723; doi:10.1038/s41598-021-96500-7)
Supplement: Supplementary file 1 — Supplementary Information 1. [file 41598_2021_96500_MOESM1_ESM.pdf]

Online Supplement

This online supplement includes detailed information and further materials on

1. the video transcription
2. the analytical sample
3. additional tables and figures
4. the survey questions used.

1 Video transcription

Good day and thank you for participating in this survey. I will walk you through the rules of an activity that you will take part in together with another randomly matched person who also participates in this survey. I will refer to this person as your partner.

You should see a table that shows different values in red and blue. These represent payoffs that you and your partner can receive in this activity in addition to your standard rewards for taking this survey. The blue values represent your payoffs, and the red values represent your partner's payoffs.

You and your partner have two options to choose from: A and B. The same table that you see on this screen will be shown to your partner. At this stage, you don't know what your partner's choice will be. Neither does your partner know what your choice will be.

Your final payment will be determined based on your and your partner's choice. You will be notified about your partner's choice and your final payoff in a couple of days. The value of the payoff will be provided through your standard reward program.

Now, let me briefly explain the different choices you have:

- If you choose option A and your partner chooses option A, you will receive **60 cents**, and your partner will also receive **60 cents**.
- If you choose option A but your partner chooses option B, you will receive **60 cents**, and your partner will receive **nothing**.
- If you choose option B and your partner chooses option A, you will receive **nothing** and your partner will receive **60 cents**.
- Finally, if you choose option B and your partner chooses option B, you will receive **1 dollar**, and your partner will also receive **1 dollar**.

To make your choice, you will need to click on one of the two buttons, either A or B, on the next screen. Your final choice cannot later be changed. Before you make a decision, I will provide you with an additional suggestion about this activity. While you have been chosen to receive this suggestion, your partner will not receive any suggestion for this activity.

Randomized advice:

1. I suggest that you select option A because it is a safe option and you will receive 60 cents no matter what your partner chooses.
2. I suggest selecting option B because you have the chance to earn the highest amount of 1 dollar if your partner also makes the same decision.

Please click on the arrow that will appear below to proceed to the next page and make your choice. You can consult the written instruction throughout the activity by using a link on the next page.

2 Sample

Our sample is based on a survey that we conducted from July 27th to August 24th, 2020. We employed the services of Qualtrics to collect a representative sample for the US population. Qualtrics based their online data collection from their pool of respondents on the following quotas:

1. Age
   - 18-24 = 13%
   - 25-34 = 18%
   - 35-44 = 17%
   - 45-54 = 18%
   - 55-64 = 16%
   - 65+ = 18%
2. Gender
   - Female = 50%
   - Male = 48%
   - Other = 2%
3. State/Region
   - Midwest = 21%
     Illinois - Indiana - Iowa - Kansas - Michigan - Minnesota - Missouri - Nebraska - North Dakota - Ohio - South Dakota - Wisconsin
   - Northeast = 18%
     Connecticut - Delaware - District of Columbia - Pennsylvania - Maine - Maryland - Massachusetts - New Hampshire - New Jersey - New York - Rhode Island - Vermont
   - South = 37%
     Alabama - Arkansas - Florida - Georgia - Kentucky - Louisiana - Mississippi - North Carolina - Oklahoma - South Carolina - Tennessee - Texas - Virginia - West Virginia
   - West = 23%
     Alaska - Arizona - California - Colorado - Hawaii - Idaho - Montana - Nevada - New Mexico - Oregon - Utah - Washington - Wyoming
4. Education
   - Less than HS diploma = 13%
   - HS diploma/GED = 28%
   - Some college (no degree) = 21%
   - Associate's degree = 8%
   - Bachelor's degree = 19%

- Graduate degree = 11%

5. Ethnicity

   - White = 62%
   - Black = 12%
   - Hispanic = 17%
   - Asian = 5%
   - Other = 4%

Recent research has shown that Qualtrics' nonprobability samples generate comparable results to conventionally sourced data [1]. Moreover, while representation is typically of highest importance for surveys, randomization is often more critical for experiments [2]. As we are testing the hypotheses based on a randomized experiment and are interested in (differences in) reactions to treatments across various groups, the representativeness of the data for the general U.S. population is less of a threat to the validity of the reported results.

The survey is composed of two modules: module 1 consists of survey questions, and in module 2, we implement our experimental design. We collect data from 2,323 respondents and assign them randomly to the control (no mask) and treatment (mask) conditions and from another 520 respondents who were not part of the control or treatment conditions. The latter sample received only written instructions instead of the video message and did not receive any suggestion about the game. The data collection was part of a larger project in which we collected a total of 5,200 responses, of which 10 percent (520) of the participants were reserved to be the opponents in the stag-hunt game. This sample allowed the stag-hunt payoff to be dependent on the opponent's decision without having the subject in each condition to consider the impact of the provided suggestion on their unknown opponent's choice.

Prior to analyzing the data, we assessed whether the targeted quotas based on age, gender, state/region, education and race/ethnicity were achieved. As we are analyzing a subsample of the total sample drawn by Qualtrics, targeted and realized shares across the quota variables may diverge and potentially bias our results. We present the achieved quotas in our sample of 2,323 respondents in Table S1, which shows some slight differences from the targeted percentages. respondents aged 25-34 years, male respondents, respondents from Western states, and respondents with less than a high school degree are underrepresented in our sample.

To assess whether our results are affected by these misrepresentations, we calculate population weights based on the Current Population Survey and the quotas provided by Qualtrics using iterative proportional fitting [3]. We employ the test proposed by DuMouchel and Duncan [4] to evaluate whether the weighted and unweighted estimates of our model are significantly different. The test, which is presented in Table S2, reveals that weighted and unweighted estimates for the treatment effect do not significantly differ. Nevertheless, in Table S4, we show that the inclusion of weights in the baseline regression does not substantially affect our estimates.

In cleaning the data, we remove (i) inconsistent responses (N=229; e.g., if the number of children in the household was larger than the overall number of people in the household), (ii) observations with missing information for one of the variables included (N=146) and (iii) outliers in regard to time it took to answer the survey. We truncated 2% of the data from the

Table S1: Achieved versus Targeted Weights

**Group**	**N**	**Achieved %**	**Targeted %**
**Age**			
18-24	365	15.71	13
25-34	369	15.88	18
35-44	375	16.14	17
45-54	374	16.1	18
55-64	412	17.74	16
65+	428	18.42	18
Total	2,323	100	100
**Gender**			
Male	978	42.1	48
Female	1343	57.81	50
Other	2	0.09	2
Total	2,323	100	100
**State/Region**			
Midwest	487	20.96	21
Northeast	489	21.05	18
South	921	39.65	37
West	426	18.34	23
Total	2,323	100	100
**Education**			
Less than high school	114	4.91	13
High school or equivalent (e.g., GED)	715	30.78	28
Some college	510	21.95	21
2-year degree (Associate's)	196	8.44	8
4-year degree (Bachelor's)	494	21.27	19
Graduate or professional degree	294	12.66	11
Total	2,323	100	100

Table S2: DuMouchel and Duncan Test

(1) population weight	=	0
(2) treatment×population weight	=	0
F(2,50)	=	0.14
Prob > F	=	0.8658

upper and lower centiles of the distribution of the total duration subjects took to complete the survey (N=81). As a result, our empirical analysis is based on 1,867 observations, where the control condition has N= 906 observations and the treatment condition has N=961 observations. Analyses using the nontruncated sample do not differ substantially in their results (see *Table S4*) but are less precise than those using the truncated sample. All 1,867 respondents answered exactly the same survey questions but differed only with regard to their assignment to the experimental conditions.

Table S3 represents a balance table that shows the number of observations, means, and standard errors across observations in the two treatment conditions for the full sample as well as the sample we use for our analysis. The table also shows whether the means in the two treatment conditions are significantly different from one another. From the table, it is clear that all the variables of interest are balanced across the individuals in the two experimental conditions, confirming that the randomization was successful and retained after the removal of observations that were insufficient.

3 Additional tables and figures

Figure S1: Generalized Trust

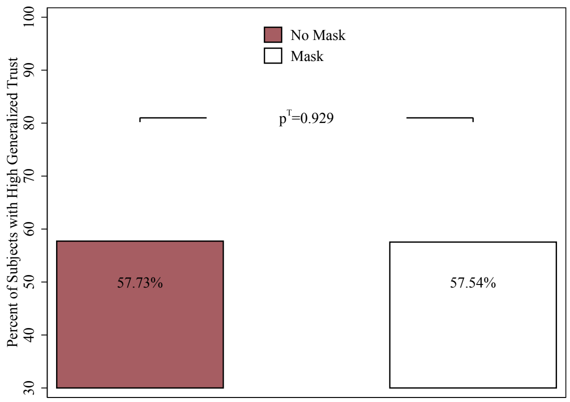

Notes: The bar in red depicts the unconditional mean in the control (no mask) condition (*N=906*), and the bar in white depicts the mean in the treatment (mask) condition (*N=961*). The p^T-value represents the p-value for the treatment effect. Standard errors are clustered at the state level.

In Figure S1, we look at whether across the treatment and control groups, there are differences in generalized trust. Generalized trust is measured using respondents' agreement with the statements: "In general, you can trust people", "Nowadays, you can't rely on anybody", and "It's better to be cautious before trusting strangers". We do not find any difference across individuals in the two conditions, which further confirms that the results are not due to generally less trusting individuals in the treatment group relative to the control group.

Table S3: Balance Table

	Full Sample					Analytical Sample				
	(1) No Mask		(2) Mask		t-test p-value	(3) No Mask		(4) Mask		t-test p-value
Variable	N/[Clusters]	Mean/SE	N/[Clusters]	Mean/SE	(1)-(2)	N/[Clusters]	Mean/SE	N/[Clusters]	Mean/SE	(1)-(2)
Above average normative beliefs	1133	0.476	1190	0.487	0.986	906	0.490	961	0.519	0.438
	[50]	[0.021]	[50]	[0.013]		[49]	[0.023]	[50]	[0.014]	
**Political Ideology**										
Liberal	1052	0.328	1099	0.338	0.474	906	0.309	961	0.319	0.477
	[50]	[0.021]	[50]	[0.022]		[49]	[0.022]	[50]	[0.022]	
Moderate	1052	0.326	1099	0.328	0.917	906	0.327	961	0.330	0.718
	[50]	[0.013]	[50]	[0.014]		[49]	[0.013]	[50]	[0.014]	
Conservative	1052	0.346	1099	0.334	0.317	906	0.364	961	0.351	0.259
	[50]	[0.020]	[50]	[0.018]		[49]	[0.022]	[50]	[0.020]	
**COVID Risk**										
High health risk	1133	0.477	1190	0.510	0.779	906	0.525	961	0.563	0.821
	[50]	[0.021]	[50]	[0.021]		[49]	[0.024]	[50]	[0.023]	
Above median age	1133	0.409	1190	0.431	0.523	906	0.464	961	0.491	0.465
	[50]	[0.021]	[50]	[0.022]		[49]	[0.022]	[50]	[0.024]	
Bad health condition	1133	0.155	1190	0.156	0.914	906	0.156	961	0.153	0.787
	[50]	[0.015]	[50]	[0.009]		[49]	[0.016]	[50]	[0.011]	
High economic risk	1133	0.170	1190	0.161	0.778	906	0.160	961	0.153	0.940
	[50]	[0.017]	[50]	[0.014]		[49]	[0.018]	[50]	[0.016]	
Unemployment	1133	0.037	1190	0.051	0.033**	906	0.034	961	0.043	0.124
	[50]	[0.007]	[50]	[0.006]		[49]	[0.008]	[50]	[0.007]	
Reduced work hours	1133	0.135	1190	0.113	0.161	906	0.128	961	0.114	0.529
	[50]	[0.014]	[50]	[0.013]		[49]	[0.014]	[50]	[0.013]	
**Design Covariates**										
Suggestion A	1133	0.508	1190	0.505	0.978	906	0.513	961	0.506	0.946
	[50]	[0.011]	[50]	[0.014]		[49]	[0.014]	[50]	[0.013]	
Norm elicitation before video	1133	0.520	1190	0.520	0.848	906	0.522	961	0.515	0.829
	[50]	[0.012]	[50]	[0.014]		[49]	[0.015]	[50]	[0.015]	
**Additional Covariates**										
Gender										
Male	1133	0.417	1190	0.425	0.913	906	0.423	961	0.435	0.706
	[50]	[0.015]	[50]	[0.017]		[49]	[0.015]	[50]	[0.019]	
Female	1133	0.583	1190	0.574	0.920	906	0.577	961	0.565	0.706
	[50]	[0.014]	[50]	[0.017]		[49]	[0.015]	[50]	[0.019]	
Other	1133	0.001	1190	0.001	0.895	906	0.000	961	0.000	N/A
	[50]	[0.001]	[50]	[0.001]		[49]	[0.000]	[50]	[0.000]	
Race										
Asian	1133	0.055	1190	0.055	0.789	906	0.051	961	0.051	0.952
	[50]	[0.012]	[50]	[0.017]		[49]	[0.013]	[50]	[0.016]	
Black	1133	0.132	1190	0.146	0.292	906	0.099	961	0.107	0.460
	[50]	[0.014]	[50]	[0.014]		[49]	[0.013]	[50]	[0.012]	
Hispanic	1133	0.080	1190	0.087	0.290	906	0.074	961	0.075	0.537
	[50]	[0.019]	[50]	[0.020]		[49]	[0.019]	[50]	[0.018]	
White	1133	0.707	1190	0.693	0.275	906	0.754	961	0.752	0.522
	[50]	[0.033]	[50]	[0.032]		[49]	[0.035]	[50]	[0.026]	
Other	1133	0.026	1190	0.019	0.469	906	0.022	961	0.015	0.330
	[50]	[0.005]	[50]	[0.004]		[49]	[0.005]	[50]	[0.003]	
Education										
Less than high school	1133	0.056	1190	0.042	0.080*	906	0.044	961	0.031	0.170
	[50]	[0.007]	[50]	[0.006]		[49]	[0.007]	[50]	[0.006]	
High school or equivalent	1133	0.290	1190	0.324	0.384	906	0.262	961	0.311	0.221
	[50]	[0.019]	[50]	[0.022]		[49]	[0.022]	[50]	[0.024]	
Some college	1133	0.233	1190	0.207	0.334	906	0.236	961	0.207	0.424
	[50]	[0.019]	[50]	[0.011]		[49]	[0.022]	[50]	[0.013]	
2-year degree (Associate's)	1133	0.084	1190	0.085	0.740	906	0.093	961	0.085	0.592
	[50]	[0.008]	[50]	[0.008]		[49]	[0.009]	[50]	[0.009]	
4-year degree (Bachelor's)	1133	0.208	1190	0.217	0.478	906	0.232	961	0.237	0.407
	[50]	[0.016]	[50]	[0.012]		[49]	[0.018]	[50]	[0.014]	
Graduate or professional degree	1133	0.128	1190	0.125	0.942	906	0.134	961	0.128	0.971
	[50]	[0.019]	[50]	[0.012]		[49]	[0.023]	[50]	[0.013]	
State and Week FE			**Yes**					**Yes**		

Note: Fixed effects for state and week are included in all estimation regressions. Standard errors are clustered at the state level. The value displayed for the t-tests are p-values. The stars indicate significance: * $p < 0.100$, ** $p < 0.050$, and *** $p < 0.010$.

Figure S2: Self-Evaluation of Trust in Video Actor

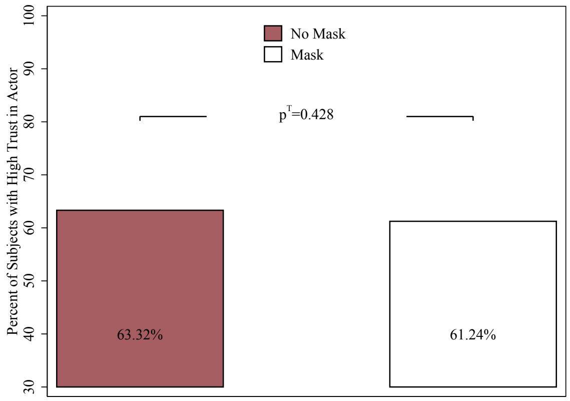

Notes: The bar in red depicts the unconditional mean in the control (no mask) condition (*N=906*), and the bar in white depicts the mean in the treatment (mask) condition (*N=961*). The p^T-value represents the p-value for the treatment effect. Standard errors are clustered at the state level.

To evaluate whether survey-based trust measures and our behavioral measure yield different results, Figure S2 shows that asking about how the respondent evaluates the actor's trustworthiness, there are no significant differences across the treatment and control groups (masked and unmasked conditions).

Table S4: Treatment effects

		**Robustness**				
	(1)	(2)	(3)	(4)	(5)	(6)
**Outcome: Follow Advice**	**Baseline**	**Less Controls**	**Logit**	**Probit**	**Weighted**	**Non-truncated Sample**
**Mask**	-0.043**	-0.041**	-0.24**	-0.14**	-0.048**	-0.038**
	(0.018)	(0.018)	(0.097)	(0.057)	(0.019)	(0.018)
State FE	*Y*	*Y*	*Y*	*Y*	*Y*	*Y*
Week FE	*Y*	*Y*	*Y*	*Y*	*Y*	*Y*
Design Controls	*Y*	*Y*	*Y*	*Y*	*Y*	*Y*
Additional Controls	*Y*	*N*	*Y*	*Y*	*Y*	*Y*
Truncation	*Y*	*Y*	*Y*	*Y*	*Y*	*N*
No. of Obs.	1867	1867	1840	1840	1867	1930

Note: Standard errors (in brackets) are clustered at the state level. The stars indicate significance: * p < 0.100 ** p < 0.050, and *** p < 0.010. *Design controls* include the suggested option during the experiment, the order of the experiment and the survey questions about personal normative beliefs. *Additional controls* include *demographic covariates*, including gender, race, and education; *personal normative beliefs*; and *COVID-related risk covariates*, including health and economic risk.

In Table S4, we present our estimates for the treatment effect in column (1). In columns (2)-(6), we show the results of the robustness analysis in which we include only the design controls, estimate a logit specification, a probit specification, a regression using survey weights and a specification without truncating the data. The estimates show that across all model specifications, there is a negative treatment effect of mask use on following advice. These results are consistent with the results based on unconditional means presented in Figure 2.

Table S5: Treatment effect by group

Outcome: Follow Advice	(1)<br>**Below vs. Above Average Normative Beliefs**	(2)<br>**Low vs. High Health Risk**	(3)<br>**High vs. Low Economic Risk**
**No Mask - Below**	*Omitted*		
**No Mask - Above**	-0.016		
	(0.028)		
**Mask - Below**	-0.087***		
	(0.031)		
**Mask - Above**	-0.016		
	(0.028)		
**No Mask - Low**		*Omitted*	
**No Mask - High**		0.033	
		(0.033)	
**Mask - Low**		-0.070**	
		(0.032)	
**Mask - High**		0.014	
		(0.036)	
**No Mask - High**			*Omitted*
**No Mask - Low**			-0.061
			(0.038)
**Mask - High**			-0.12***
			(0.042)
**Mask - Low**			-0.090**
			(0.043)
	Difference between treatment effects		
Coefficient (β^D)	0.087*	0.050	0.091**
Standard error (se^D)	(0.046)	(0.039)	(0.043)
p-value (p^D)	[0.066]	[0.20]	[0.040]
State FE	*Y*	*Y*	*Y*
Week FE	*Y*	*Y*	*Y*
Design Controls	*Y*	*Y*	*Y*
Additional Controls	*Y*	*Y*	*Y*
Truncation	*Y*	*Y*	*Y*
No. of Obs.	1867	1867	1867

Note: Standard errors (in brackets) are clustered at the state level. The stars indicate significance: * p < 0.100 ** p < 0.050, and *** p < 0.010. *Design controls* include the suggested option during the experiment, the order of the experiment and the survey questions about personal normative beliefs. *Additional controls* include *demographic covariates*, including gender, race, and education; *personal normative beliefs*; and *COVID-related risk covariates*, including health and economic risk.

In Table S5, we present our estimates for the interaction effect between the treatment and the covariates for the groups of interest. We find that groups with lower health risk (column (1)), higher economic risk (column (2)) and below-average personal normative beliefs (column (3)) have reduced interpersonal trust in people wearing a mask (treatment) versus those not wearing a mask (control). These results are consistent with the results based on conditional means presented in Figures 3-4.

4 Robustness

COVID-related risks: In the main analysis, we include a composite measure for respondents' health risk and economic risk. For the health risk, we consider two factors: age above 50 and the perception of one's own health as fair or poor. These factors are often highlighted by public health officials when categorizing individuals into risk groups for a potentially severe course of COVID-19. Analogous to the health risk measure, we use two factors – unemployment or reduced work hours experienced by the household between January and July/August – to construct an indicator measuring whether individuals are exposed to high economic risk during the COVID-19 pandemic.

Figure S3: Follow Advice by Health Risk and Economic Hardship

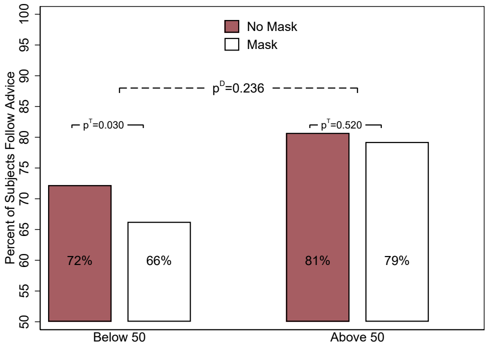

(a) Health Risk (Age)

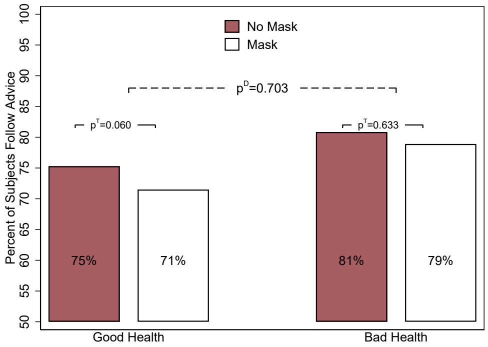

(b) Health Risk (Health Condition)

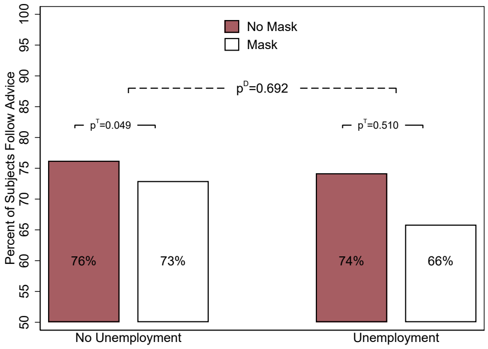

(c) Economic Risk (Unemployment)

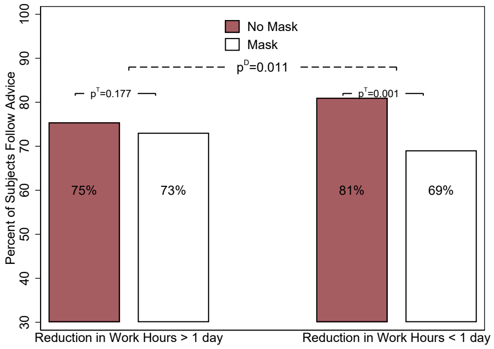

(d) Economic Risk (Reduced Work Hours)

Notes: The bar in red depicts the conditional means in the control (no mask) condition (*N=906*), and the bar in white depicts the mean in the treatment (mask) condition (*N=961*). The p^T-value represents the p-value for the treatment effect. Standard errors are clustered at the state level. Conditional means control for state fixed effects, week fixed effects, design controls and individual groups.

As an additional robustness check, we study whether the treatment effect differs for each of the two factors included in the composite measures for high and low health and economic risk. We present these results in Figure S3 and show that the composite index provides us with results very similar to the individual factors used for the construction of the index. In particular, Figure S3(a) shows that on average, mask use lowers trust among the younger population in the US: 9.09% (approx. 6 percentage points) fewer participants follow the advice given by a

stranger wearing a mask than by a stranger not wearing a mask (p^T-value = 0.030). Among the older group, the probability of following advice is not significantly affected by whether the stranger is wearing a mask. Comparing the treatment effect across the two groups, however, the difference is insignificant (p^D-value = 0.236), which suggests that age does not play a strong role defining who trusts others wearing face masks more or less.

To further explore how perceived risk changes the way mask use affects interpersonal trust, Figure S3(b) shows that wearing a mask only marginally significantly affects trust among healthier respondents (p^T-value = 0.060), while there is no significant effect among unhealthier respondents. The difference in the treatment effects again is not significant (the p^D-value is = 0.703). Similar to our main analyses, neither age nor health condition seems to play a substantial role in the effects of mask use on trust.

We next take a more detailed look at the effects of mask use on trust among those at increased economic risk and analyze their effects separately for respondents who themselves or whose partner experienced a transition to unemployment or reduced their working hours since January 2020. As Figure S3(c) shows, the effect size is largest for those whose households experienced a transition to unemployment or a reduction in working hours. However, the effect among the former is insignificant, and the difference in effects compared to those who did not transition to unemployment is also insignificant. The lack of significance is likely due to a lack of power, as only 2% of our sample reported that they themselves or their partners transitioned into unemployment between January and July/August. Many respondents reported being furloughed (and thus having reduced working hours) instead of being unemployed. Consequently, we urge caution in interpreting the effect for unemployment but highlight the alternative measure for economic risk in the form of having reduced working hours in Figure 3(d). The figure mirrors the main results presented in the paper, where wearing a mask reduces trust by 15% (approx. 12 percentage points) for the group that experienced substantially reduced working hours (p^T-value = 0.001), while there was no significant difference for the other group. The results suggest that economic risk (at least in the US) is driven primarily by a reduction in working hours (or being furloughed), while unemployment (at least in July and August) seems to play a rather marginal role.

Alternative health risk: As an alternative (and more conservative) health risk index, we categorize individuals above 60 or who perceive their health condition to be poor as our high-health-risk group (as opposed to individuals above 50 and individuals who perceive their health condition as fair or poor). This categorization presents a group that is at a higher health risk relative to the index we employed in the main analysis. In Figure S4, we show that our baseline results are consistent with the results using this alternative index for health risk.

The results show that 8% (approx. 7 percentage points) fewer individuals with a high health risk trust the advice from the masked individual than that from the unmasked individual (p^T = 0.020). We also find that 1.2% (approx. 1 percentage point) more individuals trust the advice from the masked individual than that from the unmasked individual, although the effect is insignificant. We again find that there is no significant difference in the treatment effect between the low- and high-risk groups, which is consistent with our main results and indicates that the role played by one's health risk is less significant in driving the effect of mask use on interpersonal trust.

Figure S4: Health Risk (Alternative Index)

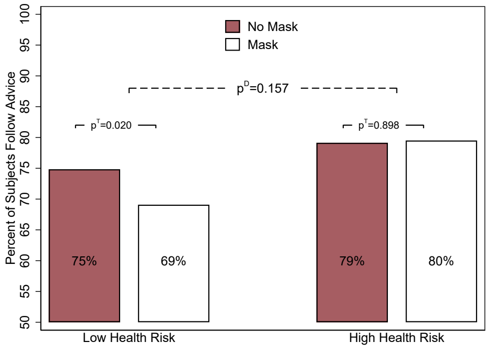

Notes: The bar in red depicts the unconditional mean in the control (no mask) condition (*N=906*), and the bar in white depicts the mean in the treatment (mask) condition (*N=961*). The p^T-value represents the p-value for the treatment effect, and the p^D-value is the p-value for the difference across the treatment effects for the mentioned groups on the x-axis. Standard errors are clustered at the state level.

Personal normative beliefs: In the main analyses, we estimate the treatment effect by below-average and above-average personal normative beliefs while controlling for state fixed effects, week fixed effects, design controls and individual characteristics. To confirm the robustness of the findings, we use alternative measures, and instead of using the composite index of COVID-related risk factors, we include (i) each of the health risk factors (dummy for the age > 50 or fair to poor health condition) separately, (ii) each of the economic risk factors (dummy for unemployment or reduced work hours) separately, and (iii) both health and economic risk factors separately.

We present the conditional means (analogous to the estimates in Table S5) in Figure S5. We find that there is (approx. 9 percentage points) less trust in the advice from an individual wearing a mask than that from the individual not wearing a mask by the group with below average personal normative beliefs about mask wearing. The effect is statistically significant ($p^T = 0.008$, 0.009, 0.008), regardless of whether we control for the disaggregating factors or the composite indexes for health and economic risk.

We furthermore include additional controls to our baseline specification. We use (i) 3 categories of liberal, moderate and conservative political ideology in Figure S6(a) and (ii) a continuous age variable in Figure S6(b). The results show that none of these additional control variables impact our baseline results presented in the main analysis.

Based on all robustness analyses, we confirm that there is a significant difference in the treatment effect between the below-average and above-average personal normative belief groups (where p^D ranges from 0.062 to 0.070). In particular, the group with below-average personal normative beliefs distrusts the masked versus non-masked individual more than the group with above-average personal normative beliefs, and this result is robust to including additional controls (such as political ideology and age) or including health and economic risk factors separately.

Figure S5: Follow Advice by Personal Normative Beliefs with Disaggregated COVID-related Risk Factors

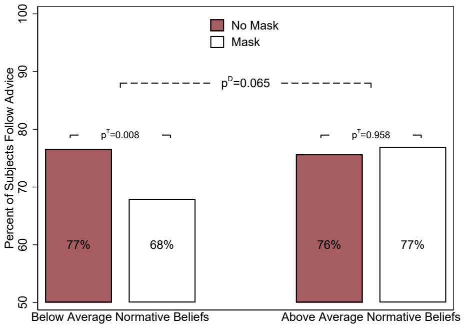

(a) Disaggregate Health Risk

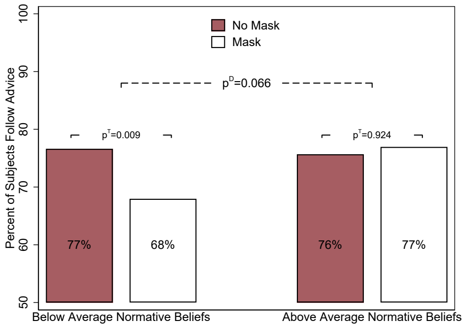

(b) Disaggregate Economic Risk

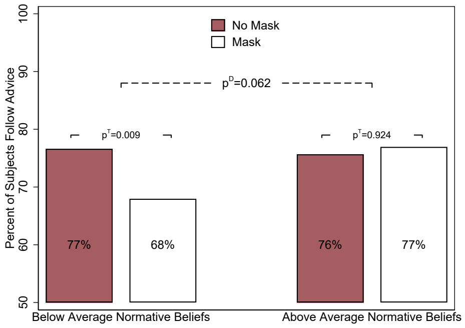

(c) Disaggregate Health & Economic Risk

Notes: The bar in red depicts the conditional means in the control (no mask) condition (*N=906*), and the bar in white depicts the mean in the treatment (mask) condition (*N=961*). The p^T-value represents the p-value for the treatment effect. Standard errors are clustered at the state level. Conditional means control for state fixed effects, week fixed effects, design controls and individual controls, along with all the group variables.

Figure S6: Follow Advice by Personal Normative Beliefs with Additional Controls

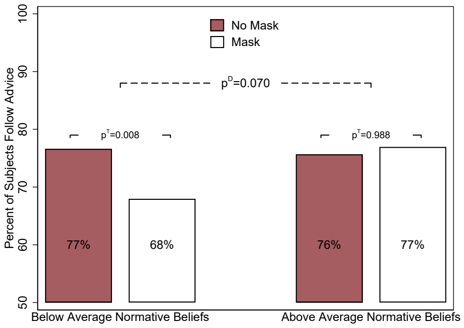

(a) With Political ideology

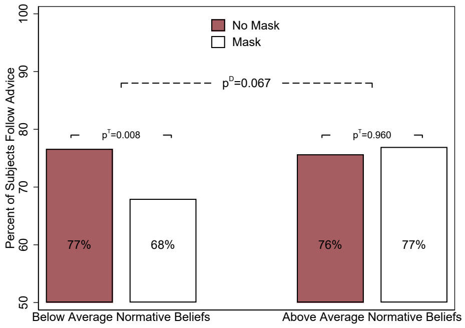

(b) With Continuous Age

Notes: The bar in red depicts the conditional means in the control (no mask) condition (*N=906*), and the bar in white depicts the mean in the treatment (mask) condition (*N=961*). The p^T-value represents the p-value for the treatment effect. Standard errors are clustered at the state level. Conditional means control for state fixed effects, week fixed effects, design controls and individual controls, along with all the group variables.

Political ideology: During the spread of COVID-19, preventive measures have evolved into a contested norm, meaning that individuals have different normative beliefs about what one should do [5]. In the US, attitudes around preventive measures are among the most heterogeneous in the world [6] Moreover, political views and ideologies shape attitudes and behavior around mask wearing [7]. Research suggests that the divide in political views about core values and beliefs hinders cross-partisan trust [8]. Political views may thus be responsible for different personal normative beliefs. We next explore the role played by these factors.

Focusing on individuals' political ideology, we find that of the three groups (liberals, moderates, and conservatives), wearing a mask significantly lowers trust only among those with a conservative ideology (see Figure S7). If advice is given by someone wearing a mask, 6.85% ($\approx$ 5 percentage points) fewer individuals follow the suggested strategy than those who follow advice given by someone without a mask (p^T-value = 0.097). Although we do not find significant treatment effects among those with a liberal or moderate ideology, the effect of political ideology is rather small, and comparing the treatment effects across the three groups, we find no significant differences. The role that political ideology plays in determining the effects of mask use on trust thus does not seem to be as strong as the role of personal normative beliefs regarding mask wearing.

Figure S7: Political Ideology

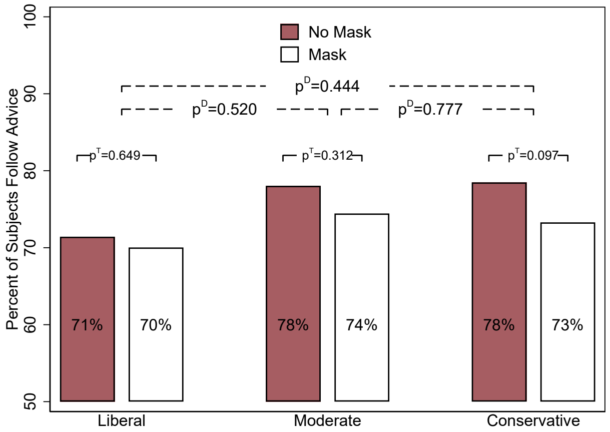

Notes: The bar in red depicts the unconditional mean in the control (no mask) condition (*N=906*), and the bar in white depicts the mean in the treatment (mask) condition (*N=961*). The p^T-value represents the p-value for the treatment effect, and the p^D-value is the p-value for the difference across the treatment effects for the mentioned groups on the x-axis. Standard errors are clustered at the state level.

5 Survey questions

Please, tell us a little bit about yourself.

What is your gender?

- ◯ Male
- ◯ Female
- ◯ Other

What is your age (in years)?

Please select your current U.S. state of residence.

- ○ Alabama
- ○ Alaska
- ○ Arizona
- ○ Arkansas
- ○ California
- ○ Colorado
- ○ Connecticut
- ○ Delaware
- ○ District of Columbia
- ○ Florida
- ○ Georgia
- ○ Hawaii
- ○ Idaho
- ○ Illinois
- ○ Indiana
- ○ Iowa
- ○ Kansas
- ○ Kentucky
- ○ Louisiana

- Maine
- Maryland
- Massachusetts
- Michigan
- Minnesota
- Mississippi
- Missouri
- Montana
- Nebraska
- Nevada
- New Hampshire
- New Jersey
- New Mexico
- New York
- North Carolina
- North Dakota
- Ohio
- Oklahoma
- Oregon
- Pennsylvania

- ○ Rhode Island
- ○ South Carolina
- ○ South Dakota
- ○ Tennessee
- ○ Texas
- ○ Utah
- ○ Vermont
- ○ Virginia
- ○ Washington
- ○ West Virginia
- ○ Wisconsin
- ○ Wyoming

What is the highest level of education you have completed?

- ○ Less than high school
- ○ High school or equivalent (e.g., GED)
- ○ Some college
- ○ 2-year degree (Associate’s)
- ○ 4-year degree (Bachelor’s)
- ○ Graduate or professional degree

What race/ethnicity do you consider yourself?

- ◯ Asian
- ◯ Black
- ◯ Hispanic
- ◯ White
- ◯ Other

Please, tell us a bit more about yourself and your household.

Are you in a relationship?

- ◯ Yes
- ◯ No

Does the person you are in a relationship with live in your household?

- ◯ Yes
- ◯ No

How many people in total are living in your household, including yourself?

- ○ 1
- ○ 2
- ○ 3
- ○ 4
- ○ 5
- ○ 6
- ○ 7
- ○ 8
- ○ 9
- ○ 10 or more

Do you have any children?

- ○ Yes
- ○ No

How many children in the following age groups are living in your household? These may or may not be your own children.

- [ ] Below the age of 5 ____________________
- [ ] Between the ages of 5 and 18 ____________________
- [ ] Above the age of 18 ____________________
- [ ] ⊗ I don't have children living in my household

There is currently a debate about when and under what circumstances one should wear a mask. Please, let us know what you think:

On a scale from -5 to 5 where -5 means "Strongly disagree" and 5 means "Strongly agree", how much do you agree with the following statements?

I think that **people should** currently **wear a mask** when

	Strongly disagree	Neither agree nor disagree	Strongly agree
	-5 -4 -3 -2 -1	0	1 2 3 4 5
**in enclosed public spaces like grocery stores**			
**outside**			
**using public transportation**			
**meeting people in public**			

You will now take part in an **activity** together **with another randomly matched person** who also participates in this survey. This person will be referred to as your partner.

You should see a **table that shows different values** in red and blue. These represent payoffs that you and your partner can receive in this activity in addition to your standard rewards for taking this survey. The **blue values represent your payoffs**, the red ones your partner's payoffs.

You and your partner have **two options to choose from: A and B**. The same table that you see on this screen will be shown to your partner. At this stage, you don't know what your partner's choice will be. Neither does your partner know what your choice will be.

Your **final payment** will be determined based on your and your partner's choice. You will be notified about your partner's choice and your final payoff in a couple of days. The value of the payoff will be provided through your standard reward program.

These are the **different choices** that you have: If you choose option A and your partner chooses option A, you will receive 60 cents and your partner will also receive 60 cents. If you choose option A but your partner chooses option B, you will receive 60 cents and your partner will receive nothing. If you choose option B and your partner chooses option A, you will receive nothing and your partner will receive 60 cents. Finally, if you choose option B and your partner chooses option B, you will receive 1 dollar and your partner will also receive 1 dollar.

To make your choice, you will need to click on one of the two buttons, either A or B, on the next screen. **Your final choice cannot later be changed.**

Please **click on the video** to **start** it. The video's duration is about three minutes.
After having watched the video, a button will appear that allows you to proceed to the next page.

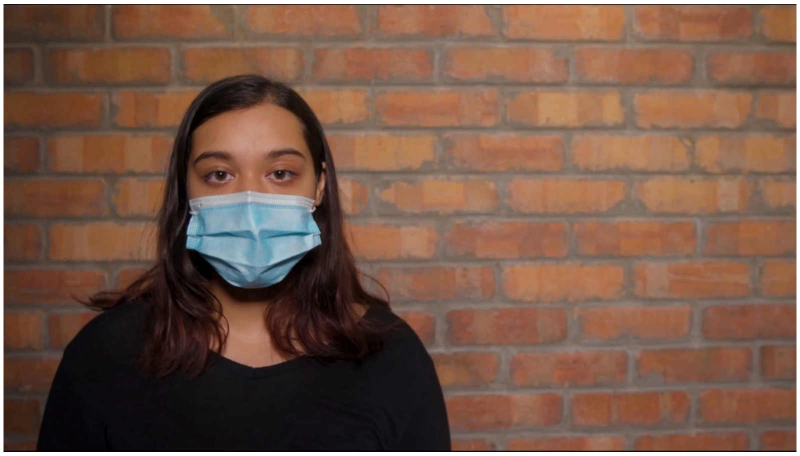

We now invite you to take part in the activity, in which you can earn **additional rewards in the value of $0 to $1.00**.

You can revisit the instructions by clicking on the button below.

		Your partner's choice	
		A	B
Your choice	A	0.6, 0.6	0.6, 0
	B	0, 0.6	1, 1

Please select what choice you would like to make.

- Option A
- Option B

Instructions

You will take part in an activity together with another randomly matched person who also participates in this survey. This person will be referred to as your partner.

You should see a table that shows different values in red and blue. These represent payoffs that you and your partner can receive in this activity in addition to your standard rewards for taking this survey. The blue values represent your payoffs, the red ones your partner's payoffs.

You and your partner have two options to choose from: A and B. The same table that you see on this screen will be shown to your partner. At this stage, you don't know what your partner's choice will be. Neither does your partner know what your choice will be.

Your final payment will be determined based on your and your partner's choice. You will be notified about your partner's choice and your final payoff in a couple of days. The value of the payoff will be provided through your standard reward program.

These are the different choices that you have:

- If you choose option A and your partner chooses option A, you will receive 60 cents and your partner will also receive 60 cents.
- If you choose option A but your partner chooses option B, you will receive 60 cents and your partner will receive nothing.
- If you choose option B and your partner chooses option A, you will receive nothing and your partner will receive 60 cents.
- Finally, if you choose option B and your partner chooses option B, you will receive 1 dollar and your partner will also receive 1 dollar.

To make your choice, you will need to click on one of the two buttons, either A or B, on the next screen. Your final choice cannot later be changed.

You have just watched a short video explaining the rules of an activity you participated in. How would you evaluate the following traits of the person who explained the rules? The person is

	Extremely	Very	Moderately	Slightly	Not at all	Don't know / Prefer not to answer
Skilled	○	○	○	○	○	○
Intelligent	○	○	○	○	○	○
Self-confident	○	○	○	○	○	○
Warm	○	○	○	○	○	○
Trustworthy	○	○	○	○	○	○

To what extent do you agree or disagree with the following statements?

	Strongly disagree	Somewhat disagree	Somewhat agree	Strongly agree
In general, you can trust people	○	○	○	○
Nowadays, you can't rely on anybody	○	○	○	○
It's better to be cautious before trusting strangers	○	○	○	○

What best describes your **current employment status**?

*If you currently work reduced hours or are on furlough, but have a valid **full-time employment contract**, please click on **full-time employed**.*

*If you currently work reduced hours or are on furlough, but have a valid **part-time employment contract**, please click on **part-time employed**.*

- ◯ Full-time employed
- ◯ Part-time employed
- ◯ Self-employed
- ◯ Unemployed
- ◯ Caregiver (e.g., children, elderly) or homemaker
- ◯ Retired
- ◯ Full-time student
- ◯ Other ____

Thinking back to January this year: was your employment status different?

- ◯ Yes
- ◯ No

During January this year what best described your employment status?

- ◯ Full-time employed
- ◯ Part-time employed
- ◯ Self-employed
- ◯ Unemployed
- ◯ Caregiver (e.g., children, elderly) or homemaker
- ◯ Retired
- ◯ Full-time student
- ◯ Other ____

Please give an estimate of your average weekly working hours.

Last week:	
During a typical week in January this year:	

Previously you told us that you are in a relationship with a person in your household. Now, we will ask some questions about this person, and refer to them as your partner.
What best describes your partner's **current employment status**?

If your partner currently works reduced hours or is on furlough, but has a valid ***full-time employment contract****, please click on* ***full-time employed****.*

If your partner currently works reduced hours or is on furlough, but has a valid ***part-time employment contract****, please click on* ***part-time employed****.*

- ◯ Full-time employed
- ◯ Part-time employed
- ◯ Self-employed
- ◯ Unemployed
- ◯ Caregiver (e.g., children, elderly) or homemaker
- ◯ Retired
- ◯ Full-time student
- ◯ Other ____

Thinking back to the end of January this year: was your partner's employment status different?

- ◯ Yes
- ◯ No

At the end of January this year what best described the employment status that your partner had?

- ○ Full-time employed
- ○ Part-time employed
- ○ Self-employed
- ○ Unemployed
- ○ Caregiver (e.g., children, elderly) or homemaker
- ○ Retired
- ○ Full-time student
- ○ Other ____

Please give an estimate of your partner's average weekly working hours.

Last week:	
During a typical week in January this year:	

On the following scale of political views ranging from extremely liberal to extremely conservative where would you place yourself?

- ○ Extremely liberal
- ○ Liberal
- ○ Slightly liberal
- ○ Moderate
- ○ Slightly conservative
- ○ Conservative
- ○ Extremely conservative
- ○ I do not wish to respond

How would you characterize your health in general?

- ○ Excellent
- ○ Very good
- ○ Good
- ○ Fair
- ○ Poor

References

[1] Sheryl L. Walter et al. “A Tale of Two Sample Sources: Do Results from Online Panel Data and Conventional Data Converge?” In: *Journal of Business and Psychology* 34.4 (2018), pp. 425–452.

[2] R. Baker et al. “Summary Report of the AAPOR Task Force on Non-probability Sampling”. In: *Journal of Survey Statistics and Methodology* 1.2 (2013), pp. 90–143.

[3] Stanislav Kolenikov. “Calibrating survey data using iterative proportional fitting (raking)”. In: *The Stata Journal* 14.1 (2014), pp. 22–59.

[4] William H DuMouchel and Greg J Duncan. “Using sample survey weights in multiple regression analyses of stratified samples”. In: *Journal of the American Statistical Association* 78.383 (1983), pp. 535–543.

[5] Cristina Bicchieri et al. “In Science we (should) trust: expectations and compliance during the COVID-19 pandemic”. In: *Preprint at Research Square* (2020), pp. 1–27. DOI: `https://doi.org/10.21203/rs.3.rs-106840/v1`.

[6] J. V. Lazarus et al. “COVID-SCORE: A global survey to assess public perceptions of government responses to COVID-19 (COVID-SCORE-10)”. In: *PLoS One* 15.10 (2020), pp. 1–18.

[7] W. Bruine de Bruin, H. W. Saw, and D. P. Goldman. “Political polarization in US residents’ COVID-19 risk perceptions, policy preferences, and protective behaviors”. In: *Journal of Risk and Uncertainty* 61 (2020), pp. 177–194.

[8] Pablo Hernandez-Lagos and Dylan Minor. “Political Identity and Trust”. In: *Quarterly Journal of Political Science* 3 (2020), pp. 337–367.
